# A Review on the Traditional Chinese Medicinal Herbs and Formulae with Hypolipidemic Effect

**DOI:** 10.1155/2014/925302

**Published:** 2014-07-07

**Authors:** Tung-Ting Sham, Chi-On Chan, You-Hua Wang, Jian-Mei Yang, Daniel Kam-Wah Mok, Shun-Wan Chan

**Affiliations:** ^1^Department of Applied Biology and Chemical Technology, The Hong Kong Polytechnic University, Hong Kong; ^2^State Key Laboratory of Chinese Medicine and Molecular Pharmacology (Incubation), Shenzhen 518057, China; ^3^Longhua Hospital, Shanghai University of Traditional Chinese Medicine, Shanghai 200032, China; ^4^Xuhui District Central Hospital of Shanghai, Shanghai 200031, China; ^5^Food Safety and Technology Research Centre, Department of Applied Biology and Chemical Technology, The Hong Kong Polytechnic University, Hong Kong

## Abstract

Hyperlipidemia, characterized by the abnormal blood lipid profiles, is one of the dominant factors of many chronic diseases such as diabetes, obesity, and cardiovascular diseases (CVD). For the low cost, effectiveness, and fewer side effects, the popularity of using traditional Chinese medicine (TCM) to handle hyperlipidemia is increasing and its role in health care has been recognized by the public at large. Despite the importance of TCM herbs and formulations, there is no comprehensive review summarizing their scientific findings on handling hyperlipidemia. This review summarizes the recent experimental and clinical results of nine representative single Chinese herbs and seven classic TCM formulae that could improve lipid profiles so as to help understand and compare their underlying mechanisms. Most of single herbs and formulae demonstrated the improvement of hyperlipidemic conditions with multiple and diverse mechanisms of actions similar to conventional Western drugs in spite of their mild side effects. Due to increasing popularity of TCM, more extensive, well-designed preclinical and clinical trials on the potential synergistic and adverse side effects of herb-drug interactions as well as their mechanisms are warranted. Hyperlipidemic patients should be warned about the potential risks of herb-drug interactions, particularly those taking anticoagulants and antiplatelet drugs.

## 1. Introduction 

Hyperlipidemia comprises a heterogeneous group of disorders, characterized by high levels in one or more lipids and/or lipoproteins [atherogenic free fatty acids (FA), triglycerides (TG) (hypertriglyceridemia), small dense low-density lipoprotein cholesterol (LDL-C) (hypercholesterolemia), and apolipoprotein (apo) B], and/or low level in antiatherogenic high density lipoprotein cholesterol (HDL-C), in the circulation [1–3]. In 2013, the American Heart Association reported that proportions of American adults aged 20 or above had abnormal blood lipid serum profile (details are shown in Table 1) [4]. These lipid disorders may occur in primary (inherited) [5–7] or secondary form [8]. Secondary hyperlipidemia arises from diet, alcohol intake, estrogen therapy, or diseases such as diabetes mellitus, hypothyroidism, erythematosus, and chronic renal diseases [9, 10]. In fact, hyperlipidemia is one of the risk factors of CVD [11], which becomes one of the major killers around the world. It was projected that by 2030 there will be about 23.3 million CVD deaths worldwide [12]. Also, CVD has imposed great medical burden to different societies around the world. The global burden of CVD is beginning to be viewed as high as infectious diseases [13]. Apart from CVD, hyperlipidemia is also closely associated with diabetes, insulin resistance, and obesity [10]. Reduction of total cholesterols (TC) and LDL-C by dietary alterations and medications that affect lipid metabolism [14] is found to reduce the occurrence of atherosclerosis in animals and clinical cardiovascular events in human [15]. Thus, prevention and treatment of hyperlipidemia are effective approaches to reduce the incident rate of chronic diseases.

Although Western medicines have been the dominant treatment used by hyperlipidemic patients, their adverse effects and part of patients' intolerance to the pharmacotherapy make traditional Chinese medicine (TCM) one of their alternatives. There is a growing interest in TCM treatment of hyperlipidemia. In the last few decades, hundreds of Chinese herbal medicines in the form of compounds, extracts, single herbs, or formulae have been reported to be effective for the prevention and treatment of hyperlipidemia [16], especially those high-fat diet (HFD) induced cases. In real practice, formulae (at least consist of two herbs) are commonly prescribed to patients. A formula generally is composed of four components: monarch, minister, assistant, and guide. The later three components aid the effects and facilitate the delivery of monarch (the principal component) or lower the toxicity of other components. Based on the patient's overall body situation, Chinese medicine practitioners always use classical formulae, rather than a single herb, as a foundation to modify the proportion and composition of the ingredients with addition or substitution by other herbs to prepare a specific formulation for individual patients.

Currently there are more than 50 TCM formulae in the form of patent drugs that have been approved by China Food and Drug Administration used for treating hyperlipidemia [16]. This implies that there is a great demand on hyperlipidemic TCM products in the market. This review summarizes TCM herbs and formulae that were proved to have effect in controlling blood lipid profiles* in vivo*,* in vitro,* and clinically so as to help people to understand and compare their underlying mechanisms. In this review, nine representative single herbs that have been studied extensively are selected (Table 2). Additionally, seven well-known TCM formulae are also covered here (Table 3).

## 2. Normal Dietary Lipid Metabolism in the Circulation

**Figure 1:**
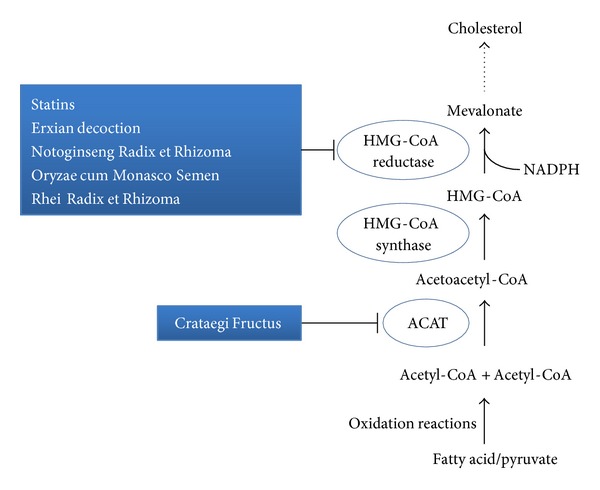
The simplified mevalonate pathway of cholesterol production. Potential therapeutic interventions in the pathway using conventional medications and TCMs are indicated. Dotted arrows: skipped pathway.

**Figure 2:**
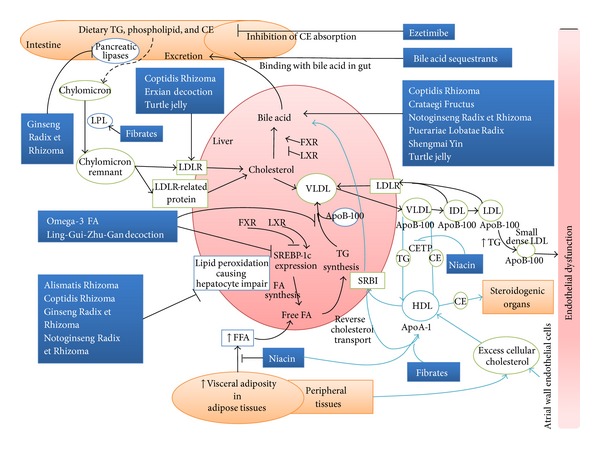
The primary pathways for the normal and abnormal metabolism of human plasma lipoproteins leading to endothelial dysfunction are summarized. Potential therapeutic interventions in the pathway using conventional medications and TCMs are indicated. Dotted arrows: skipped pathway.

Lipid metabolisms involve different lipoproteins in the anabolism and catabolism of these substances (Figures 1 to 2) [3, 9, 14, 17, 18]. TG, phospholipids, and cholesterol esters (CE) are the predominant dietary lipids. These lipids, mainly TG, are hydrolyzed by different pancreatic lipases in the intestine and then absorbed by intestinal mucosal cells and secreted into mesenteric lymphatic vessels in the form of chylomicrons with apoB-48. The newly synthesized TG and CE in the chylomicrons are hydrolyzed by lipoprotein lipase (LPL) to yield chylomicron remnant particles which are cleared by LDL receptors (LDLR) and LDLR-related proteins to the liver. The liver secretes very low-density lipoproteins (VLDL) that contain specific apoB-100, apoC-II, and apoE that bind to enzymes or receptors to facilitate the lipid transfer to the peripheral tissues including vessels for metabolism or storage. ApoB-100 is the main apolipoprotein needed for LDL uptake by the liver. TG is hydrolyzed by LPL in VLDL which is further transformed into TG-reduced intermediate-density lipoprotein (IDL) followed by LDL. LDL is recirculated into the liver or peripheral tissues [19].

HDL plays a critical role in cholesterol homeostasis to induce antiatherogenic effect, which is brought by a process called “reverse cholesterol transport” [2, 20].

The process is the opposing movement of cholesterol from peripheral cells through plasma to the liver. The process involves removal of cholesterol from arterial macrophages and peripheral cells and delivery of the excess cholesterol to the liver for excretion. HDL, containing apoA-1 and other enzymes, is synthesized by the liver in a cholesterol deplete state and takes up excess cellular cholesterol from peripheral cells and arterial macrophages. The excess cholesterol is then delivered to steroidogenic organs for hormone synthesis, or to the liver by binding to scavenger receptor class B, type I (SRBI) for further elimination into the bile as free cholesterol or as biliary acids after metabolism [21]. Liver X receptor (LXR) and farnesoid X receptor (FXR) maintain cholesterol, bile acid, and TG homeostasis [22]. This helps the removal of nonesterified free cholesterol from the blood circulation, thereby preventing the formation of arterial plaques. In indirect reverse cholesterol transport, CE is exchanged 1 : 1 for TG between apo B-containing lipoproteins (chylomicrons, VLDL, IDL and LDL) and HDL_2_ in a process regulated by the cholesterol ester transfer protein (CETP). In addition to the cholesterol removal, HDL also exerts other antiatherogenic and vascular protective functions [2] such as antioxidative [23, 24], antithrombotic [25], and anti-inflammatory actions [26].

Hence, the HDL-C level correlates inversely with the risk of CVD and atherosclerosis while high level of LDL-C highly increases their risk.

## 3. Lipid Abnormality in Hyperlipidemia Resulting in Atherosclerosis

Lipid abnormality in hyperlipidemia is an increase in nonesterified free FAs in the circulation, an inadequate esterification, and/or a reduced free FA metabolism [27]. The reduced retention of FAs by adipose tissue due to excessive visceral adiposity leads to an increased flux of free FAs through the hydrolysis of adipocyte TG, returning to the liver, which stimulates hepatic TG synthesis at hepatocytes, promoting the production of apolipoprotein B and the assembly and secretion of TG containing VLDL. When plasma TG concentration subsequently increases, TG-rich HDL-apoA-1 particles are formed and undergo catabolism. Elevated VLDL particles due to hepatic overproduction are lysed and hence fail to bind efficiently to LDLR, while the exchange of CE with TG forms TG-rich lipoproteins, resulting in the formation of abnormal small dense LDL-C particles [28, 29]. These small dense LDL particles have weaker interaction with LDLR and stay longer than normal LDL in the blood so they readily penetrate through endothelial fenestrations to accumulate in the subendothelial space [30] where they are oxidized by reactive oxidative species (ROS) such as superoxide generated by endothelial cells [31] and activated leukocytes [32]. The oxidized LDL (ox-LDL) is a key component in the development of endothelial lesion [33, 34]. It can induce endothelial expression and secretion of cytokines, growth factors, and several cell surface adhesion molecules that are capable of recruiting circulating monocytes and T lymphocytes into the intima.

**Figure 3:**
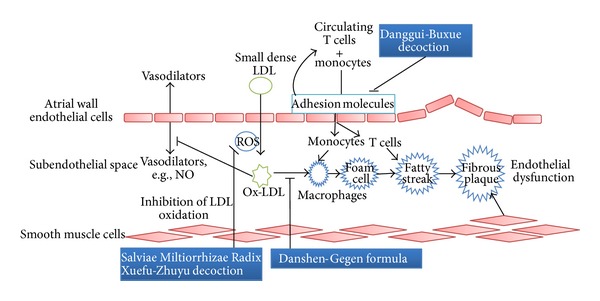
The endothelial dysfunction development is summarized and potential therapeutic interventions in the pathway using TCMs are indicated.

Ox-LDL activates the differentiation of monocytes into macrophages. It also inhibits the production of vasodilators such as nitric oxide (NO) [35] released by the endothelial cells [36]. The ox-LDL is mostly taken up by macrophage scavenger receptors [37] and accumulates to become large foam cells which become fatty streak with T cells and smooth muscles. The fatty streak then progresses into an intermediate lesion, finally into a fibrous plaque which enlarges and projects into the arterial lumen and impede the flow of blood, thus inducing atherosclerosis. Thus, a strong association exists between LDL-C levels and incidence of coronary artery diseases [38, 39].  Figure 3 summarized the development of endothelial dysfunction.

## 4. Western Medications

There are over 10 conventional and new therapies for hyperlipidemia [10], such as agonists of peroxisome proliferator-activated receptors (PPAR) [40], inhibition of cholesterol absorption with statin, fibrates [41], ezetimibe [42], bile acid sequestrants [43], niacin [44, 45], and intake of omega-3 FA [46–48]. Statins and fibrates are the most commonly used lipid-lowering medications in primary and secondary preventions of atherosclerotic disease. Although these classical hypolipidemic agents are generally well tolerated, most of them have adverse effects [49]; they only target one class of lipoproteins or may not be affordable, deterring most patients from receiving treatments [50]. For instance, statin is a group of 3-hydroxy-3-methyl-glutaryl-coenzyme A (HMG-CoA) reductase inhibitors that inhibit the mevalonate pathway so as to suppress the production of cholesterol (Figure 1). Yet, statin was reported to have possible adverse effects (myalgia, fatigue, dyspnea, memory loss, and peripheral neuropathy) [51] and a number of patients did not respond well to such therapy [52]. If patients cannot tolerate the side effects of these hypolipidemic medicines, TCM will be an ideal alternative. It is because some of the TCM herbs and formulae have been demonstrated to be effective in handling hyperlipidemia, with low cost [16, 53]. Figures 1–3 indicate the potential mechanisms of TCMs and Western drugs reported.

## 5. Studies of Single Herbs

### 5.1. Alismatis Rhizoma

Alismatis Rhizoma, also known as Zexie in Chinese, is the dried rhizome of* Alisma orientale* (Sam.) Juzep. It is to promote urination to drain dampness, discharge heat, resolve turbidity, and lower lipid recorded in Pharmacopoeia of the People's Republic of China (CP) [54]. A number of Chinese published reports have demonstrated that AR is clinically effective in the treatment of hyperlipidemia. The oral administration of aqueous and alcoholic Zexie extracts (6 g/kg/day, 2 weeks) resulted in significant decreases in serum TG and TC levels in mice fed with HFD [55, 56]. However, the mechanism had not been clearly investigated. Dan. et al. found out that Zexie treatment (2.26 g/kg/day) resulted in an obvious decrease in serum and liver cholesterol, TG level along with elevated serum HDL-C in hyperlipidemic Kunming mice [57]. These results confirmed the efficacy of Zexie in the treatment of hyperlipidemia. They suggested that the herb might act by decreasing the liver synthesis of cholesterol, rather than by increasing the cholesterol catabolism. Zexie methanolic extract (150, 300, and 600 mg/kg/day, 6 weeks) also demonstrated the prevention of the oxidative stress by lessening lipid peroxidation and activating antioxidant enzymes and markedly decreased the serum and liver lipids in nonalcoholic fatty liver disease (NAFLD) rat induced by HFD [58]. Experiments showed that the active lipid-lowering compounds are triterpenoids. Supplement of the atherogenic diet with natural alisol A-24-monoacetate isolated from Zexie in five concentrations (12.2–196.7 mg/kg/day, 10 days) lowered cholesterol levels in the plasma and liver of hypercholesterolemic rats in a dose-dependent manner [59]. Other effective alisol-triterpenes included alisol A-23, 24-diacetate (derived chemically from alisol A), natural alisol C-23-monoacetate, alisol A, and alisol B-23-monoacetate isolated from Zexie [59]. Also, no adverse effects of triterpenoid-enriched extract of Zexie (360, 720, and 1440 mg/kg/day) were observed in both genders of Sprague-Dawley (SD) rats after feeding for 90 days [60]. The adverse effects of Zexie are correlated with hepatotoxicity in chronic hepatitis B patients and nephrotoxicity following overdosage [61]. Alisol C, 16,23-oxido-alisol B, and alisol O in Zexie may cause nephrotoxicity [62].

### 5.2. Coptidis Rhizoma

Coptidis Rhizoma, commonly known as Huanglian in Chinese or Chinese Goldthread in English, is the dried rhizome of* Coptis chinensis *Franch. or* C*.* deltoidea *C. Y. Cheng et Hsiao. It is to clear heat and dry dampness, discharge fire, and remove toxin recorded in CP [54]. Different clinical,* in vivo,* and* in vitro* studies suggested multiple hypolipidemic mechanisms. Yokozawa et al. found that the herb water extract (50 and 100 mg/kg/day, 30 days) significantly reduced the levels of serum TC, LDL and ox-LDL, thiobarbituric acid-reactive substance, and liver cholesterol in a dose-dependent manner in Wistar rats but it did not reduce that of fecal cholesterol, suggesting that it reduced cholesterol synthesis by reducing lipid peroxidation [63]. Cao et al. got a consistent result as Yokozawa's but used the alkaloid extracts in HFD-fed SD rats instead [64]. The extracts (50, 100, and 200 mg/kg/day) markedly increased the level of liver total bile acid in a dose-dependent manner compared with simvastatin, which was attributed to the positive regulation of PPAR*α* and the negative modulation of FXR to upregulate the gene expression of CYP7A1 to increase its activity in the liver for cholesterol conversion into bile acids [64]. In addition, a new finding suggested that antimicrobial activities of the herb ethanol extract and berberine in HFD-fed C57BL/6J mice might also result in decreasing degradation of dietary polysaccharides, lowering potential calorie intake, and then systemically activating Fiaf protein and related gene expressions of mitochondrial energy metabolism in visceral adipose tissues [65]. Berberine is the main bioactive alkaloid of the herb. Oral administration of berberine (0.5 g twice/day, 3 months) for the hypercholesterolemic Chinese patients resulted in significant decline in serum levels of cholesterol, TG, and LDL-C but no change in HDL-C compared to placebo groups [66]. This was also replicated by another study in human subjects with a mild weight loss (average 5 lb/subject) and in SD rats [67]. The mechanism was explained by the upregulation of LDLR by berberine in the test of human hepatoma cells and the treatment of hyperlipidemic hamsters [66]. Combination of berberine with simvastatin increased the LDLR gene expression to a level significantly higher than that in monotherapies in rats and hypercholesterolemic patients [68].

Human subjects had no side effects observed and were well tolerated with the dosage of berberine [66, 67] or combined with simvastatin [68] so reduction of statin dosage in clinic by adding berberine may be possible.

### 5.3. Crataegi Fructus

Crataegi Fructus (the dried ripe fruit of* Crataegus pinnatifida* Bge. and* C*.* pinnatifida *Bge. var.* major* N. E. Br., known as Shanzha in Chinese or hawthorn in English) is a representative herb that is effective in promoting digestion and invigorating the stomach, resolving turgidity and lowering lipid, moving* Qi* and dissipating blood stasis [54]. Studies have shown that the sugar-free water extract of Shanzha was effective against hyperlipidemia by activating PPAR*α* to lower lipid levels [69]. Apart from this, the extract also inhibited atherosclerosis progression by reducing inflammatory cytokine responses and regulating endothelial function in HFD-fed rats [70]. Shanzha is rich in triterpenic acids (e.g., oleanolic acid and ursolic acid) and polyphenols (such as epicatechin, procyanidins, hyperoside, isoquercitrin, and chlorogenic acid). Oleanolic acid and ursolic acid of Shanzha were found to be particularly responsible for lowering plasma VLDL and LDL cholesterol concentrations by inhibiting intestinal acyl CoA-cholesterol acyl-transferase (ACAT, the enzyme in the mevalonate pathway to synthesize cholesterol) activity in human colon carcinoma cell line Caco-2 [71]. The blunted acetylcholine-induced, endothelium-dependent relaxation of isolated aortas of HCD-fed rats was improved by dried powder of Shanzha crude drug (20 g/kg/day, 4 weeks) [72].

The most frequent mild adverse events were dizziness, nausea, fall, gastrointestinal haemorrhage, circulation failure, and erythematous rash [73, 74] but many cases had insufficient data supplied to prove the association between the herb and specific adverse effects [75]. The herb was well tolerated and there were no reports of drug interactions [75].

### 5.4. Ginseng Radix et Rhizoma and Ginseng Radix et Rhizoma Rubra

Ginseng Radix et Rhizoma (Renshen in Chinese and Asian ginseng in English) is the dried root of* Panax ginseng *C. A. Mey. As recorded in CP, it is used for greatly tonifying the original* Qi* resuming pulse and securing collapse, replenishing “*lung*” and “*spleen*,” engendering fluid and nourishing blood, calming the mental state, and enhancing intelligence [54]. Ginseng Radix et Rhizoma Rubra, also called red ginseng, refers to the steamed and dried form of* P. ginseng* [54]. The lipid-lowering effect has been well studied in the last two decades in clinical trials [76, 77],* in vivo* [78–85], and* in vitro* [86].

For the improvement of lipid profile, the 50% alcohol herb extract (2 g three times/day, 8 weeks) decreased TC, TG, and LDL levels and increased HDL in eight normal humans compared to the blood before the trial, which might be induced by antioxidant potential of the extract as one of action mechanisms [76]. The TC and LDL-lowering result was agreed on by a research with 20 hypercholesterolemic patients at a lower dose (100 mg twice/day, 8 weeks) [77]. Yet, the patients had no change of HDL, TG, or VLDL compared to control [77]. Red ginseng saponins (0.01 g/kg for 4 weeks) sustained LPL activity at a normal level or protected LPL activity, resulting in reduction of the rise in serum TG and TC in a cyclophosphamide-induced hyperlipidemia fasted rabbit model [78]. In addition to the saponins, acidic polysaccharides (100 to 1000 mg/kg) isolated from Korean red ginseng using hyperlipidemic rats were also reported to have dose-dependently reduction in TG in serum and liver via the activation of LPL activity [81]. The hypercholesterolemia-enhanced platelet aggregation was also attenuated by Korean red ginseng, rich in glycosylated saponins (200 mg/kg/day, 8 weeks) via the suppression of diacylglycerol liberation in rabbits fed on a diet high in cholesterol, but such effect was weaker than lovastatin [79].

The antioxidant properties of enzyme induced by the herb were also not consistent in humans [76, 87] and rabbit studies [83]. The improvement of lipid peroxidation in liver by reduction in serum malondialdehyde (MDA) was generally observed in most hypolipidemic studies [76, 83, 87]. Apart from that, morphological changes of the aorta in hypercholesterolemic New Zealand white rabbits by the phenolic extract showed positive effects [83].

Weight gain reduction in 3% herb extracted saponins-containing HFD-fed male Balb/c mice may be mediated by pancreatic lipase inhibition [82]. Lin et al. suggested that intraperitoneal injections of ginsenoside Rb_1_ daily to C57BL/6 mice inhibited the desire for food intake via modifying the serum content and mRNA expression of neuropeptide Y, Y2 receptor, and peptide YY [85]. In the contrast, there were no significant body weight differences in hypercholesterolemic rabbits by the phenolic extract [83] and in HFD-fed C57BL/6 mice by fermented red ginseng powder (150 mg/kg/day, 11 weeks) [88]. However, the combination of levan (100 mg/kg/day) and the fermented red ginseng powder (150 mg/kg/day) significantly lowered body weight, serum TC and leptin levels, and fat mass with decreasing food efficiency ratio. This suggested improvement of leptin resistance associated with the obese mice. The disagreement in the hypolipidemic effect of the herb may be attributed to the composition of extract used in the experiments, administration method, dosages, and duration [83].

The safety profile of ginseng is generally good [89]. Excessive and uncontrolled intake of ginseng products may cause hypertension, nervousness, irritability, diarrhea, skin eruptions, and insomnia (collectively called ginseng abuse syndrome) [90]. More concerns about interactions between ginseng with anticoagulants, phenelzine [91], or warfarin [92] may increase the risk of bleeding episodes [93].

### 5.5. Notoginseng Radix et Rhizoma

Notoginseng Radix et Rhizoma is the rhizome and root of* Panax notoginseng *(Burk.) F. H. Chen, commonly called Sanqi in Chinese, is a herb that invigorates blood circulation and has been widely used in TCM to treat cardiovascular diseases [54, 94]. In CP, the action of Sanqi is to dissipate stasis, stanch bleeding, dispel swelling, and relieve pain [54]. Sanqi crude drug powder supplement incorporated in the HFD for SD rats (10 g/kg, 4 weeks) was found to have improvement in lipid profiles, reduction of HMG-CoA reductase level, and inhibitory effect of lipid peroxidation by increase in the activity of antioxidant enzymes [hepatic superoxide dismutase (SOD) and glutathione peroxidase] [95]. Sanqi saponins including different ginsenosides and notoginsenosides were found to be the main active compounds. A systematic study [96] reported that diet induced hypercholesterolemic SD rats fed with total saponins of Sanqi (30 or 100 mg/kg/day, 4 weeks) could significantly reduce elevated serum TC, TG, LDL-C, and atherogenic index and increase HDL-C. The saponins significantly improved the endothelium-dependent vasodilatation, acetylcholine-induced NO production, and endothelial NO synthase mRNA expression. The findings suggested that Sanqi could prevent the development of hypercholesterolemia and atherosclerosis by inducing the biosynthesis of bile acids from cholesterol and promoting the *β*-oxidation of FA in the liver. Increasing the gene expression of endothelial NO synthase in endothelial cells and Sanqi antioxidative activity might bring about its vasoprotection. Another research also proved that the saponins (100 mg/kg/day, 9 weeks) markedly reduced serum TC, TG, and blood viscosity in HFD-fed SD rats and exerted antiatherosclerosis through an anti-inflammatory action and regulation of the blood lipid profile, involving nuclear factor-*κ*B signaling pathway [97]. Another experiment using the n-butanol extract (30, 60, and 100 mg/kg/day, 4 weeks) showed similar results and revealed that the extract acted as a dual FXR/LXR*α* agonist to prevent the accumulation of abnormal lipid in the hyperlipidemic rats [98]. One study [99] concerned that high fibrinogen is an emerging independent risk factor for cardiovascular diseases but statins could only slightly reduce blood fibrinogen level. In particular, intake of atorvastatin, a very potent lipid lowering agent, might have association with increase in fibrinogenaemia in some treated patients [100, 101]. Compared to fluvastatin (3 mg/kg/day), Sanqi powder (43 and 86 mg/kg/day) showed a similar activity in decreasing plasma TC but more marked reduction in fibrinogenaemia in HFD-fed rats [99].

More awareness using the intravenous injection of Sanqi saponins should be taken since some adverse reactions such as epistasis, allergy, and even anaphylactic shock might be caused [102]. Abdominal complaints, nausea, and dyspepsia were observed clinically in a pellet consisting of extracts from Danshen, Sanqi, and Borneol [103].

### 5.6. Oryzae cum Monasco Semen

Oryzae cum Monasco Semen is commonly called as Hongqu in Chinese and red yeast rice (RYR) in English. It has long been recognized as a TCM to strengthen “*spleen*” to improve food digestion and promote blood circulation to dissipate blood stasis [94]. It is produced by solid-state fermentation of washed and cooked rice using red yeast (*Monascus purpureus* Went). RYR is well known for its blood cholesterol lowering effect. The fermentation of RYR produces a family of monacolins that resemble HMG-CoA reductase inhibitors [104]. Among these products, monacolin K is the same substance that the USA Food and Drug Administration approved as lovastatin [105]. Lots of evidences showed that RYR lowers cholesterol levels moderately compared to other statin drugs, but with less adverse effects [106–109]. One recent study demonstrated that 22 patients receiving RYR capsules that contained 7.2 mg lovastatin and a total of 2.4 mg of other monacolins experienced a significant reduction in LDL-C (23.0%) and TC (15.5%) compared to the placebo after 16 weeks of treatment [110]. Another randomized controlled trial showed that RYR preparations had lowering lipid effect, compared with placebo, statins, or other active lipid-lowering agents, and with no treatment received for 12 weeks [111]. In this test, the lipid modification effects of RYR were observed better than nicotinate and fish oils, similar to pravastatin, simvastatin, lovastatin, atorvastatin, or fluvastatin, but equal to or less effective than fenofibrate and gemfibrozil.

Dizziness and gastrointestinal discomfort were also reported in 1.3–36% of participants after RYR intakes [111]. In spite of its therapeutic effect, the yeasts from the same genus of RYR (*Monascus *strains) could produce citrinin (a nephrotoxic mycotoxin) as a secondary toxic metabolite. A survey indicated that 69.0% of red yeast rice, 35.1% of dietary supplements, and 5.7% of red yeast rice processed products contained citrinin in Taiwan from 2009 to 2012 [112]. The safety control of this kind of products should be cautious.

### 5.7. Puerariae Lobatae Radix

Puerariae Lobatae Radix (Gegen in Chinese and Kudzu root in English) is the dried root of* Pueraria lobata *(Willd.) Ohwi for resolving the flesh and reducing fever, engendering fluid to quench thirst, promoting eruption, uprasing the middle* Qi* to relieve diarrhea, unblocking meridian and activating collaterals, and removing wine toxin in CP [54]. For the hypolipidemic effect, puerarin, the major isoflavonoid compound of Gegen, (300 mg/kg/day, 4 weeks), attenuated the increased TC induced by HCD in both serum and liver of SD rats. The cholesterol lowering action might be caused by the promotion of cholesterol and bile acids excretion in liver [113]. Additionally, this herb exhibited beneficial effect in lipid metabolism in ovariectomized (OVX) rats which imitated the postmenopausal situation of the disorders of lipid metabolism. The total isoflavones inhibited the increase in body weight and lipoprotein levels in OVX rats, which exhibited most of the characteristics of human menopausal symptoms compared to the OVX-control rats and exhibited a hepatoprotective effect in OVX-induced hepatic steatosis [114]. Long-term dietary Gegen extract supplementation containing ~25.3% puerarin, 7.1% daidzin, and 0.8% daidzein (a polyphenol-free diet, with 0.2% kudzu root extract, 2 months) could improve blood glucose, lipid, and pressure control in intact and OVX stroke-prone spontaneously hypertensive rats [115]. The Gegen flavones (100 mg/kg/day, 5 weeks) demonstrated estrogen-like effect on lipid metabolism in liver and adipose tissue compared to estrogen-treated OVX Wistar rats [116].

However, the phytoestrogens of Gegen (coumestrol, genistein, and daidzein) may have potential interactions with the endocrine system, and therefore special attention should be drawn to the herbal formulae containing Gegen, especially the prescriptions for female patients at reproductive age suffering from respiratory diseases [117]. Also, a herb-drug interaction study pointed out that isoflavones and their glycosides and other polyphenols of Gegen might be transformed into conjugated metabolites in SD rats to compete with methotrexate (a drug for cancer treatment and may cause very serious, life-threatening side effects) to delay the elimination of methotrexate, increasing its life-threatening toxicity [118].

### 5.8. Rhei Radix et Rhizoma

Rhei Radix et Rhizoma (Dahuang in Chinese and rhubarb in English) comes from the root and rhizome of* Rheum palmatum* L.,* R. tanguticum* Maxim, or* R. officinale *Baill. Dahuang has been widely used for the treatment of constipation, dysentery, and jaundice [54, 119–122]. Rhein is an anthraquinone and is one of the major components of* R. palmatum* L. Rhein (150 mg/kg/day) purified from* R. tanguticum* decreased the plasma levels of cholesterols, TG, LDL-C, and Apo-E, similar to the simvastatin group (20 mg/kg/day) in db/db diabetic mice, but it had a weaker inhibitory effect on HMG-CoA reductase than simvastatin* in vitro* [123]. Rhein (150 mg/kg/day) was also found to decrease body and fat weight, lower hepatic lipid levels, improve insulin resistance, normalize alanine aminotransferase levels, and reverse hepatic steatosis on NAFLD in HFD-fed mice [124] and might protect against obesity and related metabolic disorders through LXR antagonism and regulation of uncoupling protein-1 expression in brown adipose tissues in HFD-fed mice [125]. Water extract capsules of root of* R. officinalis *(50 mg/kg/day, oral ingestion with 200 mL of water for six months) was found to significantly lower TC and LDL-C levels but had no change in TG and HDL-C levels in the clinical trial group of patients with atherosclerosis compared to the control group [126]. The researchers suggested that the change aided the improvement in the endothelial function. The same type of water extract was administrated in New Zealand white rabbits and gained similar results [127]. The extract had antiatherosclerotic and plaque stabilizing effects which might occur due to a reduction of some inflammatory cytokines activated by toll-like receptors and nuclear factor- *κ*B signaling [127].

No serious adverse effects were observed throughout the clinical trial [126]. Only five patients in the trial group reported diarrhea, which was mild and resolved without symptomatic therapy [126]. Since Rhubarb has purgative effect due to its anthraquinones [128], the major symptoms of overdose are griping and severe diarrhoea with consequent losses of fluid and electrolytes [129].

### 5.9. Salviae Miltiorrhizae Radix

Salviae Miltiorrhizae Radix (also called Danshen in Chinese), the dried root of* Salvia miltiorrhiza* Bunge, is a TCM herb commonly used for the prevention and treatment of CVD. In CP [54], it is used for promoting blood circulation and dispelling stasis, nourishing the blood and calming mental state, regulating menstruation and suppressing pains, cooling blood, and eliminating carbuncle. It has been used for treatment of CVD for long time. The cardiovascular protective effect mainly contributed by Danshen's antioxidants has been observed in many animal models. Treatment with aqueous extract of Danshen (600 mg/kg/day, 12 weeks) reduced TC and LDL-C with no change in TG and HDL-C and prevented formation of HFD induced fatty liver in an OVX hyperlipidemic rat model [130]. Oxidative stress is one of the causative factors that link hypercholesterolemia with the pathogenesis of atherosclerosis. Increased oxidative stress, resulting from increased ROS production, appears to play an important role in hypercholesterolemic atherogenesis [131]. The high antioxidative capacity of the Danshen aqueous extract may facilitate the removal of ROS in the circulation. Another study suggested that this antioxidant capacity and cholesterol lowering effect observed in the hypercholesterolemic restenosis New Zealand white rabbit model fed with 80% ethanol extract of Danshen treatment (4.8 mg/kg/day, 6 weeks) enhanced smooth muscle apoptosis and attenuates neointimal hyperplasia [132]. Same types of model fed with a HCD containing 5% water-soluble Danshen extract for 12 weeks reduced the atherosclerotic area in the abdominal aorta by 56% and cholesterol deposition in the thoracic aorta by 50%, although it was weaker than probucol treated group. It concluded that Danshen's cholesterol-lowering effect and more importantly, the antioxidant, salvianolic acid B, that accounted for about 75% of antioxidant activity in Danshen contributed to the prevention of endothelial damage and inhibition of LDL oxidation [133]. In another study, hyperlipidemic SD rats were treated with purified aqueous extracts of Danshen that contained Danshensu, rosmarinic acid, and salvianolic acid A and salvianolic acid B (50, 100, and 150 mg/kg/day, 4 weeks). They also decreased TC and TG levels and increased HDL-C serum levels, but this improvement likely contributed to the extract acting as a FXR/LXR*α* co-agonist [134].

There was low or nontoxic acute (32 g/kg, twice/day) or subchronic administrations (5.76 g/kg/day, 13 weeks) of Danshen injection in rats but a significant decrease in TG and increase in total bilirubin [135]. Coadministration of Danshen may exaggerate the anticoagulant response to warfarin since Danshen might increase the bioavailability of warfarin [136, 137]. Therefore, Danshen or its products such as Danshen-Gegen formula or Danshen dropping pills should be used under close medical supervision by people taking similar anticoagulants.

## 6. Studies of TCM Formulae

### 6.1. Danggui-Buxue Decoction

Danggui-Buxue decoction (DGBX) is a very common TCM prescription consisting of Astragali Radix and Angelicae Sinensis Radix at a dose of 1 : 5 [138]. In CP, its product “Danggui Buxue Koufuye” (a DGBX oral solution) is recorded to raise the* Qi* and nourish the blood [54]. A study used DGBX (3 or 6 g/kg/day) for 4 weeks to be orally administered to the diabetic atherosclerosis rats, which were induced by NO inhibition plus HFD [139]. Although there was no significant change in TG level, serum TC and LDL-C were significantly lowered, and the HDL-C was higher in the DGBX-treated group than that in diabetic atherosclerosis model groups. Treatment with DGBX in early diabetic atherosclerosis rats resulted in significant inhibition of mRNAs expression of monocyte chemoattractant protein-1 (MCP-1), intercellular adhesion molecule-1 (ICAM-1), and CD36 (a FA translocase that mediates oxidized LDL uptake) in aorta so it helped the prevention of diabetic atherosclerosis. Another study showed the similar effect of DGBX (1.68, 8.4 and 16.8 g/kg/day) for 5 weeks but had obvious decrease in TG in all dosages [140]. DGBX (equivalent to 6 g/kg/day, 6 weeks) also demonstrated improvement in hepatic lipid peroxidation by increase in SOD activities and decreased MDA levels in fibrotic livers of HFD-fed rats [141].

The clinical treatment of DGBX preparations at 1.5, 3.0, or 6.0 g/day, 12 weeks was well tolerated, with no serious adverse events noted in postmenopausal women but no significant lipid profile changes were observed [142].

### 6.2. Danshen-Gegen Formula

Danshen-Gegen formula (DG), composed of Salviae Miltiorrhizae Radix and Puerariae Lobatae Radix, are traditionally paired clinically to treat atherosclerosis, myocardial infarction, and other cardiac symptoms and have been widely studied in the aspect of cardiovascular effects [143–146]. Various concentrations (0.1–1.0 mg/mL) of the water extract in the ratio of 7 : 3 were tested in the human monocyte derived macrophages loaded with acetylated LDL [147]. Compared to the control, the herbal mixture induced a significant dose-dependent decrease in the (free and esterified) cholesterols in the macrophages. However, the herb pair also induced an increase in ICAM-1 expression and monocyte adhesion at higher concentrations [147].

In a clinical trial, DG treatment (3 g/day, 24 weeks) in double-blind parallel fashion in patients suffering from coronary artery disease showed mild decrease in TC and LDL-C [148]. Another clinical research with postmenopausal women treated by the herb pair (two capsules, containing 1 g water extract/day) demonstrated that the herb-pair treated group has a significant improvement in intima-media thickness and a remarkable decrease in TC and LDL as compared to placebo-treated group after 12-month treatment [149]. For the genomic study [149] the herbal-treated group has a higher number of differential gene expressions identified as compared to the placebo-treated groups. It was suggested that the herb pair could diminish the process of cardiovascular deterioration in postmenopausal women.

For the safety of DG, its treatment in patients suffering from coronary artery disease was well tolerated [148]. Yet, coadministration of DG with anticoagulant warfarin or aspirin, respectively, can decrease the prothrombin time of the drugs and cause other significant pharmacokinetic and pharmacodynamic herb-drug interactions in SD rats [150].

### 6.3. Erxian Decoction

Erxian decoction (EX), designed by Zhang Bo-Na in early 1950s, consists of Curculiginis Rhizoma, Epimedii Folium (monarch), Angelicae Sinensis Radix, Morindae Officinalis Radix (minister), Anemarrhenae Rhizoma, and Phellodendri Chinensis Cortex (assistant) without fixed ratio [151]. It has long been used in TCM to treat* Yang *and* Yin *deficiency of “*kidney,*” menopausal symptoms, and osteoporosis [152, 153]. A study demonstrated that water extract of EX (equivalent to 4.10 g crude drug/kg/day, 8 weeks) had hypolipidemic effects in a menopausal rat model but this was not observed in ethyl acetate (0.11 g/kg), n-butanol (0.470 g/kg), and the aqueous remaining fractions (2.34 g/kg) of the water extracts [154]. Premarin, which is a conjugated estrogen for the replacement of hormone of postmenopausal women, did not show such effect. The serum levels of TC and LDL-C of EX groups were suppressed, possibly through the downregulation of HMG-CoA and upregulation of the LDLR while HDL-C and TG levels had insignificant change. Compared to the premarin, EX containing hypolipidemic components may have the advantage to hyperlipidemic postmenopausal women.

In a clinical trial, the water extract of EX (equivalent to 62 g of crude herbal materials/day, 12 weeks) is well tolerated, with no serious adverse events noted in Hong Kong perimenopausal women [153].

### 6.4. Ling-Gui-Zhu-Gan Decoction

Ling-Gui-Zhu-Gan decoction (LGZG) is an ancient Chinese herbal formula from “Jin-Gui-Yao-Lue” for warming* Yang* for resolving fluid retention and strengthening “*spleen*” to resolve dampness [155]. It consists of four herbs only: Poria, Cinnamomi Ramulus, Atractylodis Macrocephalae Rhizoma, and Glycyrrhizae Radix at the weight ratio of 4 : 3 : 3 : 2 [155]. Caloric restriction therapy has been studied with metabolic disorders such as type 2 diabetes [156, 157] and obesity [158] to normalize metabolism. Recently, scientists have studied caloric restriction therapy supplemented with LGZD in Wistar rats [155]. Although there were no statistical differences in blood lipid level between fasting and those fed with LGZD groups, the blood level of ghrelin, a starvation hormone [159], was lower in HFD rat groups fasting 24 hours intermittently supplemented with LGZD than those without LGZD. Thus, it was suggested that the decoction may help regulate appetite of the fasting rats and so the same may happen in human patients for easier completion of the caloric restriction therapy when they feel less hunger [155]. Another research group provided evidence on the traditional treatment principle of LGZD using HFD induced rat models of NAFLD [160]. The possible mechanisms of LGZG to regulate lipid metabolism are to increase serum thyroid hormone levels and improve FA synthesis via modulation of thyroid hormone receptor *β*1 (TR*β*1) and carnitine palmitoyltransferase-1A (CPT1A) expression in liver and enhance metabolism and transport of FA through modulation of sterol regulatory element-binding protein 1c (SREBP-1c), long-chain acyl-CoA synthetase (ACSL), and ApoB-100 expression. The combination of Poria and Ramulus Cinnamomi might be crucial in LGZG to warm* Yang* to activate* Qi* by increasing serum thyroid hormone levels and hepatic TR*β*1 and CPT1A expression and enhancing *β*-oxidation of FA, so as to relieve water retention while Atractylodis Macrocephalae Rhizoma and Glycyrrhizae Radix assist such effect of warming* Yang*.

### 6.5. Shengmai Yin

Shengmai Yin (SMY) which is also called “Shengmai San” or “Pulse-activating decoction” is comprised of Ginseng Radix et Rhizoma, Ophiopogonis Radix, and Schisandra Chinensis Fructus (2 : 1 : 2) [161], for treating cardiovascular diseases such as coronary heart diseases [162] and brain impairment [163]. It was first documented in “Nei-Wai-Shang-Bian-Huo-Lun” in the 13th century. Its patent drug “Shenmai Capsule” and SMY are officially recorded in CP with Ginseng Radix et Rhizoma Rubra replacing unprocessed Ginseng Radix et Rhizoma instead to replenish both* Qi* and* Yin* energies, generate body fluids, and restore the pulses [54]. Some ingredients in SMY may increase the dissolution of schisandrin when decocting* in vitro* and delay its elimination and enhance its bioavailability in rat compared to the Schisandra Chinensis Fructus aqueous extract [164]. An animal study in a Chinese journal showed that Shengmaidan chewable tablets (equivalent to 2.0 g crude drug/kg, 4 weeks) could reduce TC and LDL-C in SD rat serum obviously while low dosage group (equivalent to 1.0 g crude drug/kg) could reduce TG in rat serum obviously [165]. It also decreased low-shear reduced viscosity of whole blood, plasma viscosity, haematocrit, and erythrocyte aggregation index but exhibited insignificant effect on the index of erythrocyte deformability. In another research, SMY had no significant effect on plasma lipids of Wistar rats but groups fed with HCD plus 4% SMY for four weeks had lower hepatic cholesterol and TG contents [166]. SMY had no effect on fecal cholesterol excretion but higher fecal bile acid content was observed after the SMY treatment. Increased fecal bile acid excretion (the major degradation of endogenous cholesterol process) might stimulate the biosynthesis of bile acid using cholesterol as the precursor, resulting in an increase in hepatic cholesterol catabolism and so reducing hepatic cholesterol and thereafter TG accumulation.

No adverse reactions were found in clinical trials of SMY capsule [167] or injection to patients with coronary heart disease complicated with diabetes mellitus [168] which is consistent with the review [169]. Yet, a case of a 71-year-old man indicated that an adverse interaction between warfarin and SMY resulted in cerebral bleeding [170]. Our previous mentioned ginseng, one of the main ingredients in SMY, probably contributed to the interaction. Another ingredient Schisandra Chinensis Fructus might also increase the metabolism of the coadministered warfarin [171].

### 6.6. Turtle Jelly

Turtle (tortoise) jelly, also called Gui-ling-gao in Chinese, is a popular traditional functional food in Southern China to clear heat, remove toxin, and promote urination [172]. The main ingredient is Testudinis Plastrum (tortoise shell), plus various Chinese medicinal herbs such as Smilacis Glabrae Rhizoma, Millettiae Speciosae Radix, Mesonae Chinensis Herba, and Lonicerae Japonicae Flos [173]. The plasma of diet-induced hypercholesterolemic rats fed with turtle jelly (3.3 or 10 mL/kg/day) for 30 days showed hypercholesteroleamic effect in terms of lowering the level of the serum TC and LDL, but increasing HDL level with a dose-dependent improvement on the atherogenic index [173]. These also brought the protection of endothelial dysfunction and livers. The hypercholesterolemic effect was suggested to be related to the blockage of downregulation of LDLR and phosphoenolpyruvate carboxykinase (PEPCK) mRNA and protein expressions as well as suppression of the upregulation of PPAR*α* mRNA and protein expressions in the livers in rats fed with HCD.

There are no studies reporting its safety issue. Since the content of turtle jelly is very complicated, its long-term efficacy and safety still need further investigations.

### 6.7. Xuefu-Zhuyu Decoction

Xuefu-Zhuyu decoction (XFZY) was described in an ancient book called “Yi-Lin-Gai-Cuo” in early 18th century [174] and is composed of two classic formulae (Taohong-Siwu decoction and Sini decoction): Bupleuri Chinensis Radix, Angelicae Sinensis Radix, Rehmanniae Radix, Paeoniae Rubra Radix, Carthami Flos, Persicae Semen, Aurantii Fructus Immaturus, Glycyrrhizae Radix, Platycodi Radix, Chuanxiong Rhizoma, and Achyranthis Bidentatae Radix [175]. It is a usual TCM formula to treat blood stasis in the chest region [176]. CP records its proprietary drug “Xuefu ZhuYu Jiangnang” (a XFZY oral capsule) for promoting blood circulation to remove blood stasis, moving* Qi* to relieve pain [54]. Its blood lipid lowering effect and improvement of* Qi* stagnation and blood stasis syndrome have been proved clinically and in animal researches in China [177–181].

Liu et al. [182] studied the effect of aqueous extract (8 g/kg/day) to HCD-fed Wistar rats. The aqueous fraction significantly lowered TG concentration and decreased TC/HDL-C and thromboxane/prostacyclin ratio (related to platelet coagulation). The 2.5% medium-polar and nonpolar fraction mixture from aqueous fraction (0.2 g/kg/day, 2 weeks), which exhibited comparable effect on serum lipid level and stronger potency to increase the prostacyclin secretion and significantly inhibitory effect on proinflammatory interleukin 8 production, was the antiatherogenic principle of the formula. In 2013, a NMR-based metabolomics approach was conducted to elucidate the mechanisms of XFZY on HFD induced hyperlipidemia [181]. 2.5, 5, and 10 g/kg (equivalent to crude drug mixture) of water extract of six XFZY ingredients (Bupleuri Chinensis Radix, Paeoniae Rubra Radix, Carthami Flos, Persicae Semen, Aurantii Submaturus Fructus, and Chuanxiong Rhizoma at the weight ratio of 3 : 3 : 3 : 2 : 2 : 1) were fed to HFD-induced SD rats for seven weeks. Statistical analysis of NMR spectra from blood plasma revealed that XFZY could attenuate hyperlipidemia, by partially reversing energy and lipid metabolism disturbance, decreasing the accumulation of *β*-hydroxybutyrate (ketone body) and acetyl-glycoproteins (inflammatory mediators), and enhancing glutathione biosynthesis probably due to antihyperlipidemia, antioxidative, and anti-inflammatory effects of its components.

No obvious adverse reaction was found during and after the treatment of XFZY capsules except one case reporting stomach discomfort [167, 176].

## 7. Discussion

TCMs have been used for over 2000 years in China. In the past few decades, they have drawn more and more attention worldwide to explore their new indications and test if they have potential to treat or prevent the disorders or dysfunctions that Western drugs or single compounds cannot easily regulate. One of the examples is hyperlipidemia. The hypolipidemic effects of TCMs have been extensively investigated and manifested* in vitro* and* in vivo* and clinically summarized in Tables 2 and 3.

In ancient TCM theory, the term “hyperlipidemia” does not exist but some scholars suggested that hyperlipidemia have many resemblances to the syndromes of dampness, turbid-phlegm, and blood stasis [183]. Taking turbid-phlegm as an example, excessive inner accumulation of phlegm in the body could block the circulation of blood and* Qi,* further leading to blood stasis. Patients generally have the symptoms of chest impediment, palpitations, dizziness, or even stroke in serious cases [184, 185]. Most TCM practitioners consider the deficiency of “*spleen,*” “*kidney,*” or poor movement of  “*liver*”* Qi* as the roots (the primary aspect of a disease) of hyperlipidemia while phlegm and stasis are the tips (the secondary aspect of a disease) [53, 185].

Three of the most frequently used strategies by TCM practitioners for the prescription of TCMs [16] are (1) to relieve food retention (e.g., Oryzae cum Monasco Semen, Crataegi Fructus), enhance purgative effect (e.g., Rhei Radix et Rhizoma), and eliminate dampness and water (e.g., Alismatis Rhizoma and Ling-Gui-Zhu-Gan decoction) by targeting the gastrointestinal tract, urinary, and biliary system (e.g., Turtle jelly); (2) to promote blood circulation and relieve blood stasis by improving cardiovascular system (e.g., Salviae Miltiorrhizae Radix, Notoginseng Radix et Rhizoma, and Xuefu-Zhuyu decoction); (3) to reinforce tonic effects by adjusting entire body functions (e.g., Ginseng Radix et Rhizoma, Danggui-Buxue decoction, and Shengmai Yin). Most of them may provide their effects via multiple approaches, such as red yeast rice that promotes blood circulation and strengthens “*spleen*” to improve digestions. Hence, patients with hyperlipidemia are prescribed appropriate combination of herbs according to their diagnosed syndromes so as to treat the roots well, rather than only target the tips (symptoms) only.

In addition to the long history of medical uses, in this review, preclinical researches and clinical trials provide supportive evidence to recommend the use of TCM therapy in treating hyperlipidemia.

In the Western medications, some single Western drugs clinically used alone or in coadministration with others in hyperlipidemic patients have their own adverse effects [10, 186, 187] that may not be entirely tolerated by all patients. For example, statins use may induce incidence of myopathy in high dose or in some groups of patients [188–190]. The US National Institutes of Health suggested that many patients under statin treatment alone do not achieve the LDL-C goal [186]. As for patients who want alternative medicines for the replacement of Western medications, TCM may be their choice despite reported mild adverse side effects observed in minor cases clinically. We cannot deny that there may be similar side effects of TCM sharing similar mechanisms as conventional drugs (Figures 1-2). Red yeast rice, for instance, containing monacolins that resemble HMG-CoA reductase inhibitors may have a high potential risk. However, we do not find a report about the intake of the herb causing similar adverse effect. This is greatly attributed to the variation in the drug preparation, dosage, administration period, and population differences. In addition, Chinese herbs are seldom used alone so their synergistic effect may enhance the efficacy whereas the amount of toxic chemicals is reduced by other coexisting phytochemicals [191] in the herbal mixtures. The combination of herbs that have different pharmacological activities may have better lipid-lowering effects than their individual ingredients or even comparable to single drugs probably contributed to their actions of multiple targets. Better quality control and more efficient drug preparation may enhance TCM oral bioavailability [192] with a lower dosage used. For instance, the oral bioavailability of the Sanqi saponins in crude drug may not be satisfying whereas some adverse reactions might be caused by using the intravenous injection [102]. Using chitosan as a bioadhesive material to prepare a modified Sanqi saponins tablet improved the bioavailability after oral administration for beagle dogs [193].

More importantly, herb-drug interaction has raised growing concern to scientists despite the fact that there is growing popularity of TCM as complementary medicines with Western drugs in various diseases, including hyperlipidemia, CVD, and diabetes [194, 195]. The literature also warns that there are potentially serious adverse effects and interactions between conventional therapies and TCMs [195, 196]. We found that adverse interactions exist in the conventional drugs with TCMs in the hyperlipidemic treatment while the synergistic effect of hypolipidemic activities using these two types of medicines simultaneously is also possible. For examples, the common hypolipidemic red rice yeast is likely to have drug interactions with niacin that has long been used to increase blood HDL levels. This is because the herb contains monacolins that resemble the pharmacokinetics of lovastatin which has synergistic effect with niacin. Possible adverse effects include flushing, pruritus, rash, or gastrointestinal adverse events [197]. In particular, hyperlipidemic patients who are generally suffering from CVD and taking conventional anticoagulants and antiplatelet drugs should not ignore the potential risks with TCMs [93]. Warfarin, an anticoagulant, is often found to interact with TCMs such as Salviae Miltiorrhizae Radix [136, 137] and Ginseng Radix et Rhizoma [92], increasing the bleeding risk of the patients. The TCM formulation composed of these herbs such as Shengmai Yin [170] and Danshen-Gegen formula [150] should also avoid coadministration of the anticoagulant.

In the contrast, the combination of TCM bioactive compounds and Western drugs may give better hypolipidemic activities than using them solely, such as the fermented red ginseng powder plus levan [88] and berberine from Coptidis Rhizoma with simvastatin [68]. This may reduce the dose of the single drugs supplemented with TCM products for those who do not tolerate these drugs well.

Since there are insufficient information about the effects of interactions of TCMs with the conventional hypolipidemic medications provided to the clinics and the general public, patients may underestimate or even may not be aware of the potential danger of herb-drug interactions which are similar to drug-drug interactions in terms of their effects on ADME properties [194, 198]. More investigation of herb-drug interactions* in vivo *and clinically with the main phytochemicals from the single Chinese herbs and more complicated TCM formulae is of high importance to compensate our limited knowledge of TCMs. Since little is known, Chinese medicine practitioners and clinicians should warn hyperlipidemic patients about the potential risks of herb-drug interactions of TCMs with Western drugs, particularly those taking anticoagulants and antiplatelet drugs.

Inconsistencies in the hypolipidemic effect of the herb were observed in different studies of this review. It may be attributed to the composition of TCM extract used in the experiments, administration method, dosages, animal models, and duration. Also, due to the limitation of present scientific technology, biological mechanisms of many other TCM formulae have not been completely elucidated [199]. More systematic, well-designed animal and randomized clinical studies in chronic administration with sufficient sample sizes are essential to investigate their exact mechanisms of the hypolipidemic effects, safety, and pharmacokinetics so as to give a more effective alternative to the hyperlipidemic patients.

## 8. Conclusion

Most of single herbs and formulae demonstrated the improvement of hyperlipidemic conditions with multiple and diverse mechanisms of actions in spite of their mild side effects. As more and more people tend to use TCM as an alternative medicine, more extensive, well-designed preclinical and clinical trials on the potential synergistic and adverse side effects of herb-drug interactions, as well as their mechanisms, are warranted. Hyperlipidemic patients should be warned about the potential risks of herb-drug interactions, particularly those taking anticoagulants and antiplatelet drugs.

## Figures and Tables

**Table 1:** Average percentage of abnormal blood lipid levels among Americans at age 20 or above [4].

Blood lipid serum	Total blood cholesterol level		LDL-C level	HDL-C level
Abnormal level (mg/dL)	≥200	≥240	≥130	≤40
Average percentage of Americans^#^	~42.4% men	~12.8% men	~34.4% men	~29.3% men
	~12.8% women	~13.6% women	~30.3% women	~12.6% women

^#^Americans include non-Hispanic whites, non-Hispanic blacks, and Mexican-Americans at age 20 or above.

**Table 2:** A summary of antihyperlipidemic effects of representative Chinese herbal medicines.

Chinese herbal medicines	Sources	Possible bioactive compounds	Effects mentioned in TCMs [54, 94]	Possible hypolipidemic mechanisms
Alismatis Rhizoma (Zexie)	Dried rhizome of *Alisma orientale* (Sam.) Juzep.	Alisol-triterpenes [59]	(i) Promoting urination to drain dampness (ii) Discharging heat (iii) Resolving turbidity and lowering lipid	(i) Decreasing the liver synthesis of cholesterol [57] (ii) Lessening lipid peroxidation and activating antioxidant enzymes [58]

Coptidis Rhizoma (Huanglian/Goldthread)	Dried rhizome of *Coptis chinensis* Franch. or *C. deltoidea* C. Y. Cheng et Hsiao.	Alkaloids [64], esp. berberine [66, 67]	(i) Clearing heat and drying dampness (ii) Discharging fire and removing toxin	(i) Reducing lipid peroxidation [63, 64] (ii) Upregulating PPAR*α* (iii) Negative modulation of FXR to upregulate the gene expression of CYP7A1 for cholesterol conversion into bile acids [64] (iv) Decreasing degradation of dietary polysaccharides [65] (v) Upregulation of LDLR *in vitro* and *in vivo* [66]

Crataegi Fructus (Shanzha/Hawthorn)	Dried ripe fruit of *Crataegus pinnatifida *Bge. and* C. pinnatifida* Bge. var. *major* N. E. Br.	Polyphenols [70] and triterpenic acids for example oleanolic acid and ursolic acid [71]	(i) Promoting digestion and invigorating the stomach (ii) Resolving turgidity and lowering lipid (iii) Moving *Qi* and dissipating blood stasis	(i) Activating PPAR*α* [69] (ii) Inhibiting intestinal ACAT activity in human colon carcinoma cell line Caco-2 [71]

Ginseng Radix et Rhizoma (Renshen/Asian ginseng)	Dried root of *Panax ginseng* C. A. Mey.	(i) Saponins [78, 79] (ii) Acidic polysaccharides [81] (iii) Phenolic extract [83]	(i) Greatly tonifying the original *Qi* (ii) Resuming pulse and securing collapse (iii) Replenishing “*lung*” and “*spleen*” (iv) Engendering fluid and nourishing blood (v) Calming the mental state and enhancing intelligence	(i) Improving lipid peroxidation in liver by reduction in serum MDA [76, 83, 87] (ii) Activating LPL activity [81] (iii) Inhibiting pancreatic lipase [82] (iv) Inhibiting food appetite via modifying the serum content and mRNA expression of neuropeptide Y, Y2 receptor, and peptide YY [85]

Notoginseng Radix et Rhizoma (Sanqi)	Rhizome and root of *Panax notoginseng* (Burk.) F. H. Chen	Sanqi saponins [95]	(i) Dissipating stasis and stanching bleeding (ii) Dispelling swelling and relieving pain	(i) Reducing HMG-CoA reductase (ii) Reducing lipid peroxidation by increase in the activity of antioxidant hepatic SOD and glutathione peroxidase [95] (iii) Inducing the biosynthesis of bile acids from cholesterol and promoting the *β*-oxidation of FA in the liver [96] (iv) Acting as a dual FXR/LXR *α* agonist [124]

Oryzae cum Monasco Semen (Red yeast rice/Hongqu)	Washed and cooked rice fermented with *Monascus purpureus* Went	Monacolins, esp. monacolin K [104, 110]	(i) Strengthening “*spleen*” to improve food digestion (ii) Promoting blood circulation to dissipate blood stasis	Containing a family of monacolins that resemble HMG-CoA reductase inhibitors [104]

Puerariae Lobatae Radix (Gegen/Kudzu root)	Dried root of *Pueraria lobata* (Willd.) Ohwi	Puerarin [113]	(i) Resolving the flesh and reducing fever (ii) Engendering fluid to quench thirst (iii) Promoting eruption (iv) Uprasing the middle *Qi* to relieve diarrhea (v) Unblocking meridian and activating collaterals (vi) Removing wine toxin	(i) Promoting cholesterol and bile acids excretion in liver [113] (ii) Estrogen-like effect on lipid metabolism in liver and adipose tissues [116] (iii) Hepatoprotective effect in OVX-induced hepatic steatosis [114]

Rhei Radix et Rhizoma (Dahuang)	Root and rhizome of *Rheum palmatum* L., *R*. *tanguticum* Maxim or *R*. *officinale *Baill.	Rhein [123–125]	(i) Relaxing the bowels (ii) Cooling the blood and removing toxin (iii) Expelling stasis to unblock the meridian	(i) Inhibitory effect on HMG-CoA reductase [123] (ii) Having LXR antagonism and regulation of uncoupling protein-1 expression in brown adipose tissues [125]

Salviae Miltiorrhizae Radix (Danshen)	Dried root of *Salvia miltiorrhiza* Bunge.	(i) Danshensu (ii) Rosmarinic acid (iii) Salvianolic acid A and B [133]	(i) Promoting blood circulation and dispelling stasis (ii) Nourishing the blood and calming mental state (iii) Regulating menstruation and suppressing pains (iv) Cooling blood and eliminating carbuncle	(i) Containing antioxidant (esp. salvianolic acid B) for prevention of endothelial damage and inhibition of LDL oxidation [133] (ii) Acting as a FXR/LXR *α* coagonist [134]

**Table 3:** A summary of antihyperlipidemic effects of different TCM formulae.

TCM formulae	Herbs (weight ratio in dose if applicable)	Effects mentioned in TCMs	Effects on blood lipid profile	Possible hypolipidemic mechanisms
Danggui-Buxue decoction	Astragali Radix and Angelicae Sinensis Radix (1 : 5) [153]	(i) Raising the *Qi* (ii) Nourishing the blood [17]	(i) Lowering serum TC and LDL-C levels (ii) Increasing HDL-C level (iii) No significant difference in TG level as compared with diabetic atherosclerosis model group [49, 154]	Downregulating the mRNA expression of MCP-1, ICAM-1, and CD36 [49, 154]

Danshen-Gegen formula	Salviae Miltiorrhizae Radix and Puerariae Lobatae Radix (7 : 3) [53]	(i) Promoting blood circulation (ii) Removing blood stasis [200]	(i) A significant dose-dependent decrease in free and esterified TC in the human monocyte derived macrophages *in vitro* [147] (ii) Lowering TC and LDL-C levels mildly in patients suffering from coronary artery disease compared with placebo [56] (iii) Lowering TC and LDL levels in postmenopausal women with hypercholesterolemia [52]	Dose-related suppression of acetylated LDL uptake by human macrophages [53]

Erxian decoction	Curculiginis Rhizoma, Epimedii Folium, Angelicae Sinensis Radix, Morindae Officinalis Radix, Anemarrhenae Rhizoma, and Phellodendri Chinensis Cortex (no fixed ratio) [60]	(i) Warming “*kidney*” *Yang* (ii) Nourishing “*kidney*” *Yin* (iii) Clearing ministerial fire (iv) Harmonizing thoroughfare and conception vessels (v) Balancing *Yin-Yang* [60]	(i) Suppressing serum TC and LDL-C levels (ii) No significant effect on HDL-C and TG levels in a menopausal rat model [54]	(i) Downregulating HMG-CoA (ii) Upregulating the LDL receptor [54]

Ling-Gui-Zhu-Gan decoction	Poria, Cinnamomi Ramulus, Atractylodis Macrocephalae Rhizoma, and Glycyrrhizae Radix (4 : 3 : 3 : 2) [63]	(i) Warming *Yang* for resolving fluid retention (ii) Strengthening the “*spleen”* to resolve dampness [63]	(i) Reducing TG and TC levels in HFD induced rat models of NAFLD [57] (ii) Lowering ghrelin level in HFD rat groups fasting intermittently supplemented with LGZD [63]	(i) Increasing serum thyroid hormone levels [57] (ii) Improving *β*-oxidation via modulation of TR*β*1 and CPT1A expression in liver [57] (iii) Enhancing metabolism and transport of FA through modulation of SREBP-1c, ACSL and ApoB100 expression [57]

Shengmai Yin	Ginseng Radix et Rhizoma, Ophiopogonis Radix, and Schisandra Chinensis Fructus (2 : 1 : 2) [58]	(i) Replenishing both *Qi* and *Yin* energies (ii) Generating body fluids (iii) Restoring the pulses [17]	(i) No significant effect on lipids of Wistar rats fed with HCD (ii) Lowering hepatic cholesterol and TG contents (iii) No effect on fecal cholesterol excretion but higher fecal bile acid content [59]	(i) Stimulating the biosynthesis of bile acid using cholesterol as the precursor (ii) Increasing hepatic cholesterol catabolism [59]

Turtle jelly	Testudinis Plastrum, plus various Chinese medicinal herbs such as Smilacis Glabrae Rhizoma, Millettiae Speciosae Radix, Mesonae Chinensis Herba, and Lonicerae Japonicae Flos [173]	(i) Clearing heat (ii) Removing toxin (iii) Promoting urination [51]	(i) Lowering serum TC and LDL levels (ii) Increasing HDL level diet-induced hypercholesterolemic SD rats [50]	(i) Blocking the downregulation of LDLR and PEPCK mRNA and protein expressions (ii) Suppressing the upregulation of PPAR*α* mRNA and protein expressions in the liver [173]

Xuefu-Zhuyu decoction	Bupleuri Chinensis Radix, Angelicae Sinensis Radix, Rehmanniae Radix, Paeoniae Rubra Radix, Carthami Flos, Persicae Semen, Aurantii Fructus Immaturus, Glycyrrhizae Radix, Platycodi Radix, Chuanxiong Rhizoma, and Achyranthis Bidentatae Radix [61]	(i) Promoting blood circulation to remove blood stasis (ii) Moving *Qi* to relieve pain [17]	(i) Lowering serum TC and LDL-C levels (ii) Increasing HDL-C level (iii) No significant difference in TG level in HFD fed SD rats as compared with the model group [62] (iv) Lowering TG level and TC/HDL-C ratio in HCD fed Wistar rats [182]	(i) Reversing energy and lipid metabolism disturbance (ii) Decreasing the accumulation acetyl-glycoproteins (iii) Enhancing glutathione biosynthesis [62] (iv) Inhibiting proinflammatory interleukin 8 production [182]

## Abbreviations

ACAT: Acyl CoA-cholesterol acyl-transferase
ACSL: Long-chain acyl-CoA synthetase
Apo: Apolipoprotein
CE: Cholesterol esters
CVD: Cardiovascular diseases
CP: Pharmacopoeia of the People's Republic of China
CPT1A: Carnitine palmitoyltransferase-1A
FA: Fatty acids
FXR: Farnesoid X receptor
LXR: Liver X receptor
HCD: High-cholesterol diet
HDL-C: High density lipoprotein cholesterol,
HFD: High-fat diet
HMG-CoA: 3-Hydroxy-3-methyl-glutaryl-coenzyme A
ICAM-1: Intercellular adhesion molecule-1
LDL-C: Low-density lipoprotein cholesterol
LDLR: LDL receptors
LPL: Lipoprotein lipase
MCP-1: Monocyte chemo-attractant protein-1
MDA: Malondialdehyde
NAFLD: Nonalcoholic fatty liver disease
NO: Nitric oxide
OVX: Ovariectomized
Ox-LDL: Oxidized LDL
PEPCK: Phosphoenolpyruvate carboxykinase
PPAR: Peroxisome proliferator-activated receptors
ROS: Reactive oxygen species
SD: Sprague-Dawley
SOD: Superoxide dismutase
SREBP-1c: Sterol regulatory element-binding protein 1c
TC: Total cholesterols
TCM: Traditional Chinese medicine
TG: Triglycerides
TR*β*1: Thyroid hormone receptor *β*1
VLDL: Very low-density lipoproteins.
